# Evaluating the cost of self-care: Impact of enhanced morbidity management protocol on people living with lymphatic filariasis in Bangladesh

**DOI:** 10.1371/journal.pntd.0014812

**Published:** 2026-10-21

**Authors:** Tijana Williams, Mohammad Jahirul Karim, Samid Siddiqui, AKM Fazlur Rahman, Salim Chowdhury, Sharmin Jahan, Sultan Mahmood AMS, Shaun P. Forbes, Hannah Betts, Mark J. Taylor, Louise A. Kelly-Hope

**Affiliations:** 1 Department of Tropical Disease Biology, Centre for Neglected Tropical Diseases, Liverpool School of Tropical Medicine, Liverpool, United Kingdom; 2 Filariasis Elimination and STH Control Programme, Communicable Disease Control, Directorate General of Health Services, Ministry of Health and Family Welfare, Dhaka, Bangladesh; 3 Centre for Injury Prevention and Research, Dhaka, Bangladesh; 4 Center for Evidence Synthesis in Health, Brown University School of Public Health, Providence, Rhode Island, United States of America; 5 Institute of Infection, Veterinary and Ecological Sciences, University of Liverpool, Liverpool, United Kingdom; Zhejiang Wanli University, CHINA

## Abstract

**Background:**

Lymphatic filariasis (LF) causes chronic lymphoedema requiring long-term self-care, often at personal cost. In post-elimination settings such as Bangladesh, understanding the financial burden of self-care is critical for sustaining morbidity management and disability prevention (MMDP). This study evaluated self-care costs and the impact of an enhanced morbidity management protocol among people affected by filarial lymphoedema in Bangladesh.

**Methodology:**

A longitudinal cohort study was conducted in Lalmonirhat and Thakurgaon districts (2020–2021). People affected by mild, moderate, and severe lymphoedema (n = 290) were assessed at baseline, trained in an enhanced self-care protocol, and assessed at a 3-month follow-up and a final follow-up conducted at 6 months in Lalmonirhat and 9 months in Thakurgaon. Data were collected on clinical characteristics (lymphoedema stage, acute attacks, pain), self-care items (soap, cream, pain relief), related costs, and adherence.

**Results:**

At baseline, participants with mild (11.2–54.7%), moderate (21.9–39.5%), and severe (23.4–49.3%) lymphoedema were included, reflecting variation across districts. Acute attacks were reported by 62.8–79.6% of participants and severe pain by 40.6–54.6%. Following the intervention, acute attacks decreased to 25.2–35.2% and severe pain to 22.4–28.0% (p < 0.001). Baseline monthly total self-care expenditure ranged from 195.3–215.2 BDT (US$2.29–2.53), with participants with severe lymphoedema incurring significantly higher costs than those with mild disease in both districts (234.3 vs 159.2 BDT in Lalmonirhat and 234.2 vs 172.0 BDT in Thakurgaon; both p < 0.05). Total monthly expenditure remained broadly stable over time, but spending shifted significantly towards soap and cream, while expenditure on pain relief decreased (p < 0.001). Exploratory mixed-effects analyses supported these findings, showing that higher expenditure was independently associated with follow-up time, district and greater baseline lymphoedema severity.

**Conclusions:**

Enhanced self-care training was associated with reductions in reported acute attacks and pain and changes in spending towards preventive self-care practices but did not eliminate the ongoing financial burden of lymphoedema, particularly for individuals with severe disease. Exploratory adjusted analyses supported the descriptive findings, indicating that expenditure was influenced by follow-up time, district and disease severity. Sustainable MMDP programmes should prioritise equitable access to essential self-care supplies to reduce out-of-pocket costs and support long-term adherence.

## Author summary

Lymphatic filariasis is a disabling neglected tropical disease that can cause chronic swelling of the limbs, known as lymphoedema. In many low-income settings, people affected rely on lifelong self-care at home—washing affected limbs, applying creams, and managing pain—often at their own expense. This study followed 290 people living with limb lymphoedema in two rural districts of northern Bangladesh, where access to health services is limited and household resources are constrained. Participants received training in an enhanced self-care protocol and were followed over a period of 6 months in Lalmonirhat and 9 months in Thakurgaon. Changes in clinical outcomes (disease stage, acute attacks, and pain) and monthly spending on key self-care items were recorded.

Following training, participants reported reduced pain and fewer acute attacks. Although total monthly spending on self-care remained broadly similar over time, spending patterns shifted. Expenditure on soap and cream—core components of effective self-care—increased, while spending on pain relief declined. People with more severe lymphoedema consistently faced higher self-care costs than those with milder disease, highlighting the disproportionate financial burden experienced by those with advanced disease.

These findings highlight a critical challenge: while enhanced self-care was associated with improved health outcomes, it does not remove the financial burden of living with lymphoedema. Even low-cost items such as soap and cream can present barriers to sustained self-care. These findings suggest that national programmes and donors should consider ensuring affordable or subsidised access to essential self-care supplies, particularly for those most severely affected.

## Introduction

Lymphatic Filariasis (LF) is a neglected tropical disease (NTD) that causes significant physical and psychological disability, particularly through lymphoedema, which can develop into painful mild, moderate and severe stages, recurrent acute dermatolymphangioadenitis (ADLA); known as acute attacks [1]. The Global Programme to Eliminate Lymphatic Filariasis (GPELF) promotes not only mass drug administration (MDA) to interrupt transmission but also morbidity management and disability prevention (MMDP), to improve the outcomes of people living with LF [1]. The World Health Organization (WHO) guidelines recommend a package of essential care, including limb hygiene, skincare, exercises, management of acute attacks, and mental health support, designed to reduce the frequency of secondary bacterial infections or acute attacks to prevent disease progression [1]. A recently developed enhanced self-care protocol shows additional benefits from MMDP [2–4].

There are an estimated 14–16 million people living with LF-related conditions worldwide with the greatest burden in Africa and South Asia [1]. Bangladesh had a high burden at the start of its national LF elimination programme in 2000 and has successfully eliminated LF as a public health problem, validated by WHO in 2023 [5]. Bangladesh has also made significant progress on MMDP, by establishing a national database of people living with lymphoedema and hydrocoele, and scaling up self-care training across many endemic districts, supported by national guidelines and health worker training [6]. A key challenge in maintaining MMDP is that lymphoedema care depends heavily on patient-led self-care, which can carry a significant personal cost, particularly for individuals with limited income or reduced ability to work due to their condition [7,8].

Understanding the self-care items that people living with lymphoedema regularly purchase, such as soap, antibacterial and/or antifungal creams and pain relief medication, is crucial for identifying where additional support may be needed [1]. This knowledge can help determine if targeted training could reduce unnecessary expenditure or help people living with LF better prioritise what they buy. It could also improve estimates of the direct costs associated with lymphoedema self-care, enabling more accurate assessments of the economic burden and potential cost-effectiveness of interventions [9]. Moreover, if people with different stages of disease severity have varying care needs, this could further inform how to optimise support. While Bangladesh has made substantial progress in scaling up MMDP training, routine self-care for lymphoedema is largely financed out-of-pocket by affected households, with limited evidence on the magnitude or distribution of these costs. This can have an impact in Bangladesh where the average monthly income in 2022 was estimated to be around US$90 and in rural areas US$72, per month based on national survey data [10].

Previous studies have highlighted the financial strain faced by households managing chronic lymphoedema. For example, Mathew et al. (2020) estimated that the annual economic burden per chronic LF case – including productivity loss and treatment costs - can exceed US$100 per year, placing a considerable burden on low-income families [8]. Studies in India have shown that both acute attack episodes and chronic conditions are associated with increased household expenditure due to treatment seeking costs, particularly expenditure on doctors’ fees, medications, as well as productivity losses associated with the conditions [7,11–13]. These recurring costs, though modest in absolute terms, can accumulate over time and be prohibitive for the most affected households. Reduced work capacity and income are key components of the economic burden of LF, contributing to household economic vulnerability [8]. It may also affect caregivers’ productivity as they must take time off work [14,15].

In Bangladesh, the most LF endemic areas are in the northern Rangpur Division, which is also one of the most socio-economically disadvantaged regions [16,17]. LF is caused by the parasitic nematode *Wuchereria bancrofti* and is transmitted by *Culex* mosquitoes that breed commonly in polluted, organic water in urban and rural settings [18]*.* In 2016, an estimated 8,681 people were living with lymphoedema in this division, with the highest concentration of cases in Lalmonirhat and Thakurgaon districts [6]. These two districts represent long-standing LF hotspots with high morbidity burdens. A recent study in Bangladesh indicates that implementing the enhanced self-care protocol can improve patient outcomes and reduce clinical symptoms [3]. While Bangladesh has shown leadership in scaling up LF morbidity mapping and MMDP activities [6], there is a gap in understanding out-of-pocket costs faced by people living with LF, particularly in a post-elimination context. The WHO MMDP manual highlights the need to strengthen patient-centred care and explicitly recommends the integration of morbidity management into routine health systems to ensure sustainability and equity [1]. Understanding these costs can help inform improvements to national MMDP activities and support effective healthcare integration and sustainable targets aligned with the WHO NTD road map for 2021–2030 [19].

This study evaluated out-of-pocket self-care expenditure and expenditure patterns among people living with lymphoedema at three time points including i) before, ii) at 3-months and iii) at 6 months in Lalmonirhat and 9 months in Thakurgaon after the training in an enhanced self-care protocol [2]. Specifically, the objectives were to:

1 Describe the clinical characteristics of lymphoedema relevant to understanding patterns of self-care use and costs.
2 Determine the out-of-pocket costs and self-care items used by people living with lymphoedema following training in the enhanced self-care protocol.
3 Examine the relationship between lymphoedema characteristics and the type of items and associated costs of self-care.
4 Explore if adherence to the enhanced self-care protocol influences the type and cost of lymphoedema self-care items.

## Methods

### Ethics statement

This study was approved by the Bangladesh Medical Research Council (BMRC) (Registration No. 313 17 06 2020) and Liverpool School of Tropical Medicine (LSTM) Research Ethics Committee (REC 20–008). Written informed consent was obtained from all participants prior to data collection. For participants who had limited formal education and were nonliterate, consent was documented in the presence of an impartial witness. All participants were made aware that participation was voluntary and that they could withdraw at any stage without penalty.

### Study design

A prospective longitudinal cohort field-based intervention study was conducted over 6 months in Lalmonirhat and 9 months in Thakurgaon located in the Rangpur Division, between October 2020 and May 2021. The costs of self-care and the impact of enhanced self-care protocol (the intervention) for people affected by lymphoedema were evaluated at three time points including i) before training in the self-care protocol (baseline) and at ii) at 3-months following the training and iii) at 6-months in Lalmonirhat and at 9 months in Thakurgaon (delay caused by COVID-19) following the training.

This study was part of a larger programme examining changes in clinical conditions using thermal imaging following the initial pilot study, the results of which will be published elsewhere. Study districts were selected using national programme data, reflecting areas with high lymphoedema burden and long-standing MMDP implementation. The sample size was 146 per district, with power set at 85%, a significance level of 5%, and an additional 25% to account for loss to follow-up.

### Setting

The study locations were selected based on national patient mapping and programmatic data between 2013 and 2016, which showed that Rangpur Division had the highest lymphoedema burden. Lalmonirhat district recorded 5583 lymphoedema cases (418.4 cases per 100,000) and Thakurgaon district recorded 2767 lymphoedema cases (194.3 per 100,000) [6]. Lymphoedema in these areas is strongly associated with long-term disability, and socio-economic and environmental factors such as rural residence, poverty, lack of sanitation, and low temperatures during the rainy season. Fig 1 highlights the location of Rangpur Division and the high number and prevalence of lymphoedema cases in the two study districts, adapted from Karim et al. (2019) [6].

**Fig 1:**
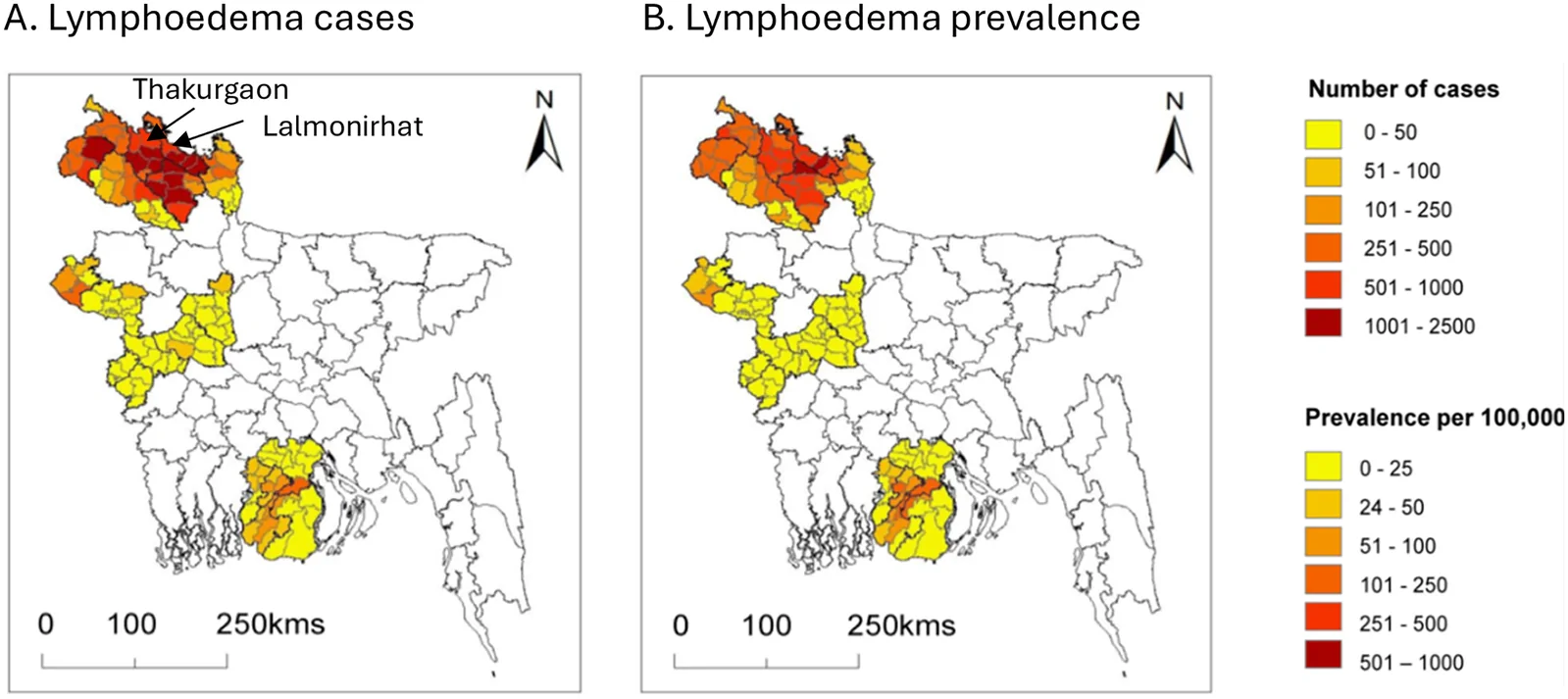
Lalmonirhat and Thakurgaon districts in Bangladesh. Note: Subset of maps adapted from Fig 2 in Karim et al, 2019.

### Population and sample size

Participants were recruited from the Bangladesh National LF Programme registry (2013–2016) [6]. In each district, 10–12 community clinics were identified, and approximately 10–15 individuals with lymphoedema were identified and invited to participate per clinic. All eligible individuals identified during recruitment agreed to participate in the study. For analysis, participants were classified into mild, moderate, and severe lymphoedema aligned to WHO/Dreyer staging, using the stage of the worst-affected leg [1,20].

### Data collection and variables

In each district, the community clinic staff/health workers identified people affected by lymphoedema, who were visited at their home and invited to participate in the study. The variables collected included demographic information; clinical condition characteristics; training in lymphoedema self-care (i.e., standard and enhanced), and items and cost of care, which are summarised below. Monthly expenditure reflected participant-reported spending during the previous month and excluded the self-care kit provided at baseline. Data collected was captured electronically using Open Data Kit (ODK) on mobile devices [21]. All data were password protected and only accessible by the research team.

Demographic variables: The participant’s age (number), sex (male, female) and marital status (married, widowed, or other) were included.

Clinical condition characteristics: The participant’s lymphoedema stage, presence of acute attacks, and pain due to lymphoedema were assessed.

Lymphoedema stage was based on the WHO categories of mild, moderate and severe severity which are broadly aligned with Dreyer’s seven stages: Mild (Dreyer Stage 1–2), Moderate (Dreyer stage 3–4), and Severe (Dreyer Stage 5–7): Further details of the staging classification are provided in S1 Text. Lymphoedema severity was assessed separately for each leg. For analysis, the higher of the two leg-stage scores (worst-affected leg) was used to represent each participant’s overall disease severity, reflecting maximum disease burden.

The presence or absence of acute attacks (ADLA- Acute dermatolymphangioadenitis) was documented and defined as by sudden episodes of fever, and skin inflammation (redness, heat, tenderness), caused by bacterial infections entering through skin lesions or cracks often lasting 2–7 days.

Pain due to lymphoedema was measured at the 3-month and 6–9 month follow-up using three questions that were part of an adapted version of a LF Quality of Life Questionnaire (LFSQQ) [22]. Participants were asked if they experienced pain when i) sitting or lying still ii) moving around the house doing everyday activities and iii) walking (e.g., to work or market). Pain levels were assessed using a self-reported scale from 1 to 5, where 1 indicated none, 2 mild, 3 moderate, 4 severe, and 5 most severe.

#### Items and cost of care.

The items used for lymphoedema self-care in the last month and their associated costs were also recorded. Items were categorised as soap, cream (antibacterial and/or antifungal), pain relief or other. The estimated cost of each item was recorded in Bangladeshi Taka (BDT) and converted to US dollars using an average 2021 exchange rate (US$1 = 85.1 BDT). The items and estimated costs did not include the standard wash kit that was given to the participants following the training at baseline.

#### Training in lymphoedema self-care.

Previous training (at baseline): Participants were asked if they had previously received training in lymphoedema self-care for their condition: yes/no.

Enhanced-care protocol: The components of the WHO-recommended self-care protocol and the enhanced self-care protocol are below:

**Table:** 

**WHO-recommended self-care**	**Additional enhanced self-care components**
Limb hygiene and washing	Self-lymphatic massage
Skin and wound care	Deep-breathing exercises
Exercise and movement	Hydration advice
Limb elevation	Nutritional advice
Management of acute attacks (ADLA)	

Participants received training in an enhanced self-care protocol that incorporated WHO-recommended self-care practices [1] and additional lymphatic-stimulating activities described by Douglass et al [2]. The enhanced protocol comprised five daily self-care activities: washing the affected limb with soap and water, applying antibacterial and/or antifungal cream, raising the affected limb, performing prescribed exercises, and massage. Participants received training at baseline and were assessed at the 3-month and final month follow-ups. They were asked if they conducted the enhanced self-care activities in the last month (Yes/No).

Further details of both protocols are available in S1 Text.

#### Adherence to the enhanced-care protocol.

Adherence to the enhanced self-care protocol was assessed at the 3-month and final follow-up visits using participants’ reported frequency of performing each of the five recommended self-care activities during the previous month. Participants who reported performing all five activities daily were classified as fully adherent. Participants who provided complete responses but reported performing one or more activities less frequently than daily were classified as partially adherent. Participants with missing responses for one or more activities were excluded from adherence analyses. Adherence was analysed as a binary outcome (fully adherent versus partially adherent). The proportions of participants classified as fully or partially adherent at each follow-up assessment are presented in S1 Fig.

### Data analysis

Descriptive statistics were used to summarise participant demographics, clinical characteristics, monthly self-care costs and adherence across the three study timepoints (baseline, 3 months and 6–9 months). Continuous variables are presented as means and standard deviations (SD), and categorical variables as frequencies and percentages. Differences in mean monthly self-care costs by lymphoedema stage, acute attack status, and pain category at baseline were analysed using one-way analysis of variance (ANOVA) or independent-samples t-tests, as appropriate. Longitudinal comparisons of monthly self-care costs between baseline and follow-up among participants with complete data were assessed using paired t-tests. Changes in categorical clinical outcomes (acute attacks and pain categories) across time points were assessed using Pearson's chi-square tests or Fisher's exact tests where expected cell counts were less than five. Results are presented as means, standard deviations (SD), percentage changes, and corresponding p-values.

Statistical significance was defined as p < 0.05. All analyses were conducted using Microsoft Excel (Microsoft Corporation, 2024) and R statistical software (Version 4.3.1).

#### Exploratory multivariable analyses.

As an additional exploratory analysis, a linear mixed-effects model was used to examine factors associated with monthly self-care expenditure while accounting for repeated observations within participants. A harmonised expenditure outcome was used, comprising monthly expenditure on soap, cream and pain relief in both districts. Total monthly expenditure was log-transformed using log(cost + 1) to account for positive skewness. Fixed effects included assessment time point, district, baseline lymphoedema stage, age, sex, previous exposure to self-care training, and an interaction between district and time point. Participant was included as a random intercept. Model coefficients were exponentiated and reported as adjusted cost ratios with 95% confidence intervals. Exponentiated coefficients are presented as adjusted cost ratios (aCRs), representing multiplicative differences in expected expenditure after adjustment for covariates. These ratios should not be interpreted as direct percentage changes in raw observed expenditure. Reference categories were Lalmonirhat (district), baseline (assessment time point), mild lymphoedema, male sex, and no previous self-care training. Model assumptions were assessed using residual-versus-fitted and normal Q–Q plots, and a sensitivity analysis excluded one observation with an absolute standardised residual greater than 3. To assess whether the district-by-time interaction improved model fit, a reduced model without the interaction was also fitted using the same analytical dataset. Model fit was compared using the Akaike information criterion (AIC), Bayesian information criterion (BIC), log-likelihood and a likelihood-ratio test.

As a second exploratory analysis, a mixed-effects logistic regression model was used to examine factors associated with adherence to the enhanced self-care protocol at post-intervention follow-up.

The analysis included the 3-month and final follow-up assessments (6 months in Lalmonirhat and 9 months in Thakurgaon). The binary outcome was coded as fully adherent versus partially adherent. An initial model included follow-up time point, district, baseline lymphoedema stage, age, sex, previous exposure to self-care training and a district-by-time interaction, with participant included as a random intercept to account for repeated observations. A reduced model without the interaction was also fitted using the same analytical dataset. Model fit was compared using AIC, BIC, log-likelihood and a likelihood-ratio test. As inclusion of the interaction did not improve model fit, the more parsimonious model without the district-by-time interaction was retained as the preferred adherence model. Results are presented as adjusted odds ratios (aORs) with 95% confidence intervals.

These multivariable analyses were considered exploratory and not prespecified; therefore, results should be interpreted cautiously and as hypothesis-generating rather than confirmatory. Models were assessed for singularity, and participant-level random-intercept variance was examined.

## Results

### Summary of study period and participant characteristics

Field surveys were conducted between October 2020 and April 2021 in Lalmonirhat district and between April and November 2021 in Thakurgaon district. At baseline, a total of 290 people affected by lymphoedema were included across the two districts. In Lalmonirhat, 152 participants were included at baseline, 151 at 3 months and 143 at 6 months. In Thakurgaon, 138 participants were included at baseline, 136 at 3 months and 125 at 9 months; the final follow-up was delayed because of the COVID-19 pandemic. Overall attrition was 1.0% at 3 months and 7.6% at final follow-up. This corresponded to follow-up completion rates of 99.0% at 3 months and 92.4% at final follow-up. Table 1 summarises baseline participant characteristics before training in the enhanced self-care protocol. Reasons for loss to follow-up were not systematically recorded.

**Table 1:** Summary of participant characteristics at baseline by district.

	Characteristics	Lalmonirhat District (n = 152)	Thakurgaon District (n = 138)
		Number of participants (%)	Number of participants (%)
**Demographic**	**Sex**		
	Male	36 (23.7)	49 (35.5)
	Female	116 (76.3)	89 (64.5)
	**Age group**		
	18-30	1 (0.7)	0 (0.0)
	30-39	0 (0.0)	6 (4.3)
	40-49	12 (7.9)	23 (16.7)
	50-59	38 (25.0)	43 (31.2)
	≥60	101 (66.4)	66 (47.8)
	**Marital status**		
	Married	95 (62.5)	114 (82.6)
	Widowed	55 (36.2)	22 (15.9)
	Other (divorced/single)	2 (1.3)	2 (1.4)
**Clinical characteristic**	**Lymphoedema stage**		
	Mild	12 (7.9)	76 (55.1)
	Moderate	54 (35.5)	30 (21.7)
	Severe	86 (56.6)	32 (23.2)
	**Acute attacks**		
	No	31 (20.4)	51 (37.0)
	Yes	121 (79.6)	87 (63.0)
	**Pain**		
	Mild	2 (2.0)	15 (10.9)
	Moderate	65 (42.8)	67 (48.6)
	Severe	84 (54.6)	56 (40.6)
**Training**	**Previous training**		
	Yes	64 (42.1)	64 (46.4)
	No	88 (55.3)	74 (53.6)
	**Enhanced care training**		
	Yes	0 (0.0)	0 (0.0)
	No	152 (100.0)	138 (100.0)

Note: Percentages are based on participants with available data for each characteristic. Baseline pain data in Thakurgaon were available for 32 participants.

#### Demographic characteristics.

In Lalmonirhat, 76.3% of the participants were female, and 66.4% of the population was over 60 years of age; most were married or widowed.

In Thakurgaon, 64.5% were female and 47.8% aged 60 years or older. Most participants were married or widowed in both districts, although greater proportion were married in Thakurgaon than in Lalmonirhat.

#### Clinical conditions.

In Lalmonirhat, 7.9% of participants had mild lymphoedema, 35.5% had moderate lymphoedema and 56.6% had severe lymphoedema at baseline. Acute attacks were reported by 79.6% of participants, and 54.6% reported severe pain.

In Thakurgaon, 55.1% had mild lymphoedema, 21.7% had moderate lymphoedema and 23.2% had severe lymphoedema. Acute attacks were reported by 63.0% of participants. Among the 32 participants with available baseline pain data, 68.8% reported severe pain.

#### Training.

In Lalmonirhat, 42.1% of participants reported previous exposure to any self-care training.

None of the participants had been exposed to the enhanced self-care protocol at baseline.

In Thakurgaon, 46.4% of participants reported previous exposure to any self-care training.

None of the participants had been exposed to the enhanced self-care protocol at baseline.

### Objective 1 – Clinical characteristics of lymphoedema

Clinical characteristics at baseline, 3 months and final follow-up are summarised by district in Table 2. Fig 2 presents changes in the proportions of participants reporting acute attacks and severe pain across the three assessment time points.

**Table 2:** Summary of clinical characteristics at baseline, 3 months, and final follow-up (6 months in Lalmonirhat and 9 months in Thakurgaon) by district.

	Lalmonirhat			Thakurgaon		
Stage of disease (n, %)	BaselineN = 152	3 monthsn = 151	6 monthsn = 143	BaselineN = 138	3 monthsn = 136	9 monthsn = 125
Mild	12 (7.9)	13 (8.7)	16 (11.2)	76 (55.1)	72 (54.5)	61 (49.6)
Moderate	54 (35.5)	52 (34.9)	53 (37.1)	30 (21.7)	32 (24.2)	34 (27.6)
Severe	86 (56.6)	84 (56.4)	74 (51.7)	32 (23.2)	28 (21.2)	28 (22.8)
**Acute Attacks (n, %)**						
No	31 (20.4)	101 (67.8)	107 (74.8)	51 (37.0)	122 (91.7)	81 (64.8)
Yes	121 (79.6)	48 (32.2)	36 (25.2)	87 (63.0)	11 (8.3)	44 (35.2)
**Pain (n, %)**						
Mild	3 (2.0)	1 (0.7)	8 (5.6)	15 (10.9)	7 (5.3)	20 (16.0)
Moderate	65 (42.8)	73 (49.0)	94 (65.7)	67 (48.6)	78 (58.6)	76 (60.8)
Severe	84 (55.3)	75 (50.3)	41 (28.7)	56 (40.6)	48 (36.1)	29 (23.2)

**Note:** Values are numbers and column percentages based on participants with valid data for each clinical outcome at each time point. Analytical denominators may therefore be lower than the total number surveyed. Valid denominators for stage were 152, 149 and 143 in Lalmonirhat and 138, 132 and 123 in Thakurgaon; for acute attacks and pain, denominators were 152, 149 and 143 in Lalmonirhat and 138, 133 and 125 in Thakurgaon.

**Fig 2:**
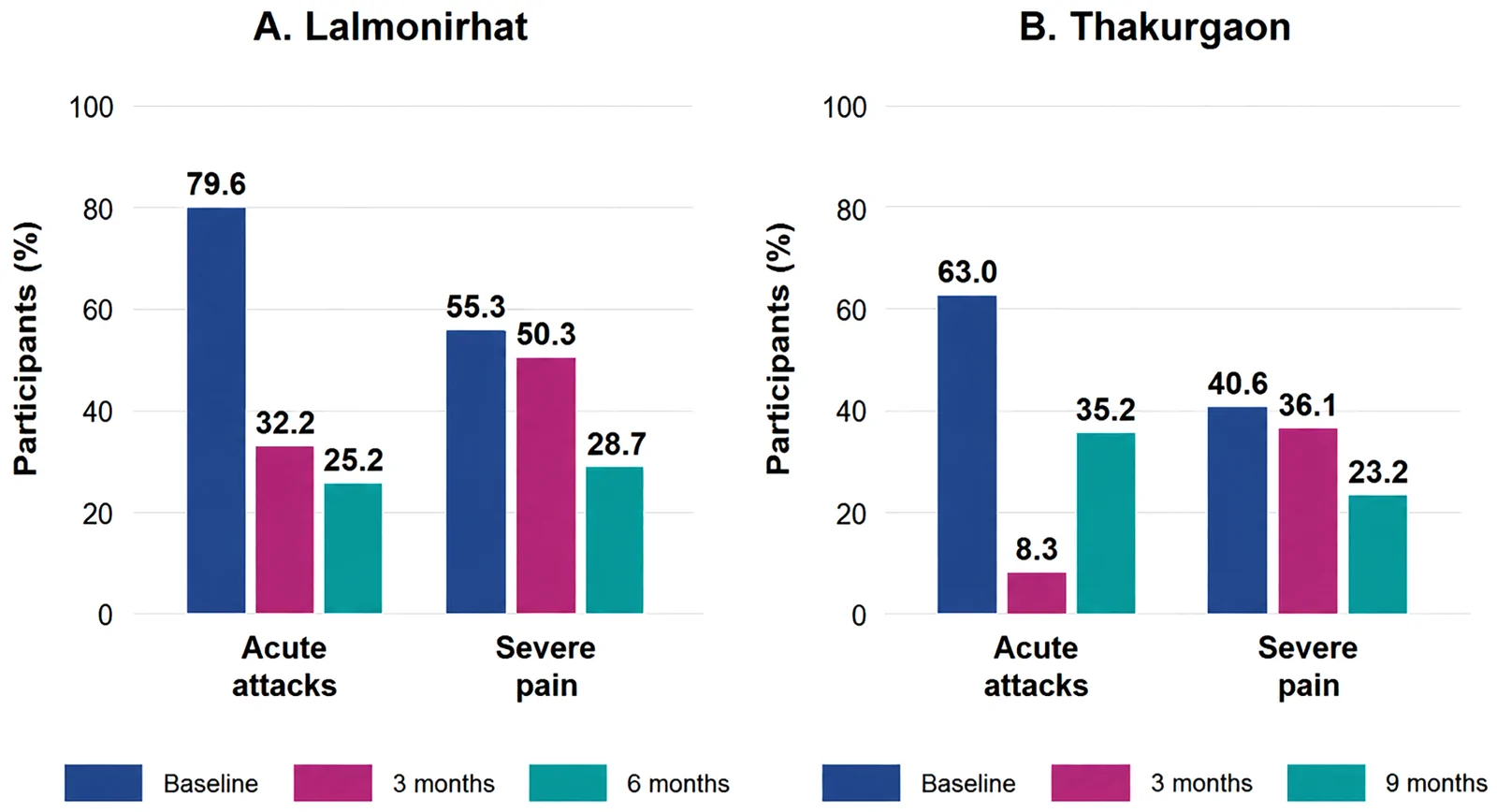
Percentage of participants reporting acute attacks and severe pain at baseline, 3 months, and final follow-up (6 months in Lalmonirhat; 9 months in Thakurgaon). Note: Values represent the percentage of participants reporting acute attacks or severe pain at each survey timepoint. Acute attacks and severe pain decreased significantly over time in both districts. Data correspond to Table 2.

#### Stage of disease.

In Lalmonirhat, the proportion of participants with severe lymphoedema remained similar between baseline and 3 months, at 56.6% and 56.4%, respectively, before decreasing to 51.7% at 6 months. The proportion with mild lymphoedema increased from 7.9% at baseline to 11.2% at 6 months.

In Thakurgaon, mild lymphoedema remained the most common stage, although its prevalence decreased from 55.1% at baseline to 49.6% at 9 months. The proportions with moderate and severe lymphoedema at 9 months were 27.6% and 22.8%, respectively.

#### Acute attacks.

In Lalmonirhat, the proportion of participants reporting acute attacks decreased from 79.6% at baseline to 32.2% at 3 months and 25.2% at 6 months. In Thakurgaon, the proportion decreased from 63.0% at baseline to 8.3% at 3 months, before increasing to 35.2% at 9 months; this remained below the baseline proportion.

Changes over time were statistically significant in both districts (p < 0.001)

#### Pain levels.

In Lalmonirhat, the proportion of participants reporting severe pain decreased from 55.3% at baseline to 50.3% at 3 months and 28.7% at 6 months. In Thakurgaon, severe pain decreased from 40.6% at baseline to 36.1% at 3 months and 23.2% at 9 months. Correspondingly, the proportion reporting mild or moderate pain increased over follow-up in both districts.

### Objective 2 - Items and cost of lymphoedema self-care

The types of self-care items used and mean monthly expenditure at baseline, 3 months and final follow-up are summarised in Table 3. Changes in mean expenditure by item category are presented graphically in S2 Fig.

**Table 3:** Mean monthly expenditure on lymphoedema self-care items at baseline and the 3-month and final follow-up, by district.

Item	Statistic	Lalmonirhat District			Thakurgaon District		
		Baseline N = 152	3 months n = 151	6 months n = 143	Baseline N = 137	3 months n = 136	9 months n = 125
**Soap**	mean	61.9	71.3	89.7	65.7	92.9	95.6
	sd	21.8	17.5	12.7	18.4	16.1	7.4
**Cream**	mean	100.1	120.1	113.4	60.6	89.9	125.1
	sd	52.6	33.6	26.5	33.7	30.9	36.9
**Pain relief**	mean	53.2	39.5	25.2	28.2	22.6	18.6
	sd	37.9	20.4	15.2	20.1	17.2	16.2
Miscellaneous*	mean	–	–	–	46.1	30.0	1.2
	sd	–	–	–	45.9	41.9	13.4
**Total**	mean	215.2	230.8	228.3	195.3	235.4	240.6
	sd	88.1	48.5	37.2	67.5	65.1	48.6

*Miscellaneous costs were not collected for Lalmonirhat. Note: Values are presented as mean monthly expenditure in Bangladeshi Taka (BDT), with standard deviation (SD), among participants with available expenditure data at each time point. Total self-care expenditure was calculated as the sum of soap, cream and pain-relief expenditure in Lalmonirhat, and these items plus miscellaneous expenditure in Thakurgaon. Clinic-visiting expenditure was not included. Miscellaneous expenditure was not collected in Lalmonirhat. Currency conversion used the 2021 exchange rate of US$1 = 85.1 BDT.

#### Overall monthly self-care costs.

In Lalmonirhat, mean monthly total self-care expenditure was 215.2 BDT (US$2.53) at baseline, 230.8 BDT (US$2.71) at 3 months and 228.3 BDT (US$2.68) at 6 months. In Thakurgaon, mean monthly total self-care expenditure was 195.3 BDT (US$2.29) at baseline, 235.4 BDT (US$2.77) at 3 months and 240.6 BDT (US$2.83) at 9 months. In analyses restricted to participants with expenditure data available at both baseline and final follow-up, the change over time was not statistically significant in Lalmonirhat (p = 0.485) but was statistically significant in Thakurgaon (p < 0.001).

#### Soap costs.

In Lalmonirhat, mean monthly expenditure on soap increased from 61.9 BDT (US$0.73) at baseline to 71.3 BDT (US$0.84) at 3 months and 89.7 BDT (US$1.05) at 6 months. This represented a 44.9% increase between baseline and 6 months.

In Thakurgaon, mean monthly expenditure on soap increased from 65.7 BDT (US$0.77) at baseline to 92.9 BDT (US$1.09) at 3 months and 95.6 BDT (US$1.12) at 9 months. This represented a 45.5% increase between baseline and 9 months.

#### Cream costs.

In Lalmonirhat, mean monthly expenditure on cream increased from 100.1 BDT (US$1.18) at baseline to 120.1 BDT (US$1.41) at 3 months, before decreasing slightly to 113.4 BDT (US$1.33) at 6 months. This represented a 13.3% increase between baseline and 6 months.

In Thakurgaon, mean monthly expenditure on cream increased from 60.6 BDT (US$0.71) at baseline to 89.9 BDT (US$1.06) at 3 months and 125.1 BDT (US$1.47) at 9 months. This represented a 106.4% increase between baseline and 9 months.

#### Pain relief costs.

In Lalmonirhat, mean monthly expenditure on pain relief decreased from 53.2 BDT (US$0.63) at baseline to 39.5 BDT (US$0.46) at 3 months and 25.2 BDT (US$0.30) at 6 months. This represented a 52.6% decrease between baseline and 6 months.

In Thakurgaon, mean monthly expenditure on pain relief decreased from 28.2 BDT (US$0.33) at baseline to 22.6 BDT (US$0.27) at 3 months and 18.6 BDT (US$0.22) at 9 months. This represented a 34.0% decrease between baseline and 9 months.

#### Miscellaneous costs.

Miscellaneous expenditure was not collected in Lalmonirhat. In Thakurgaon, mean monthly miscellaneous expenditure decreased from 46.1 BDT (US$0.54) at baseline to 30.0 BDT (US$0.35) at 3 months and 1.2 BDT (US$0.01) at 9 months. This represented a 97.4% decrease between baseline and 9 months.

### Objective 3 - Relationship between lymphoedema characteristics and the type of items and associated costs of self-care

Associations between baseline clinical characteristics and monthly self-care expenditure are summarised separately for Lalmonirhat and Thakurgaon in S1 and S2 Tables, respectively. Expenditure is presented for soap, cream, pain relief, miscellaneous items where collected, and total self-care expenditure.

Fig 3 presents changes in mean expenditure on soap, cream and pain relief from baseline to 3 months and final follow-up among participants with severe lymphoedema, those reporting acute attacks, and those reporting severe pain. Across these selected clinical groups, expenditure on soap and cream generally increased over time, while expenditure on pain relief decreased.

**Fig 3:**
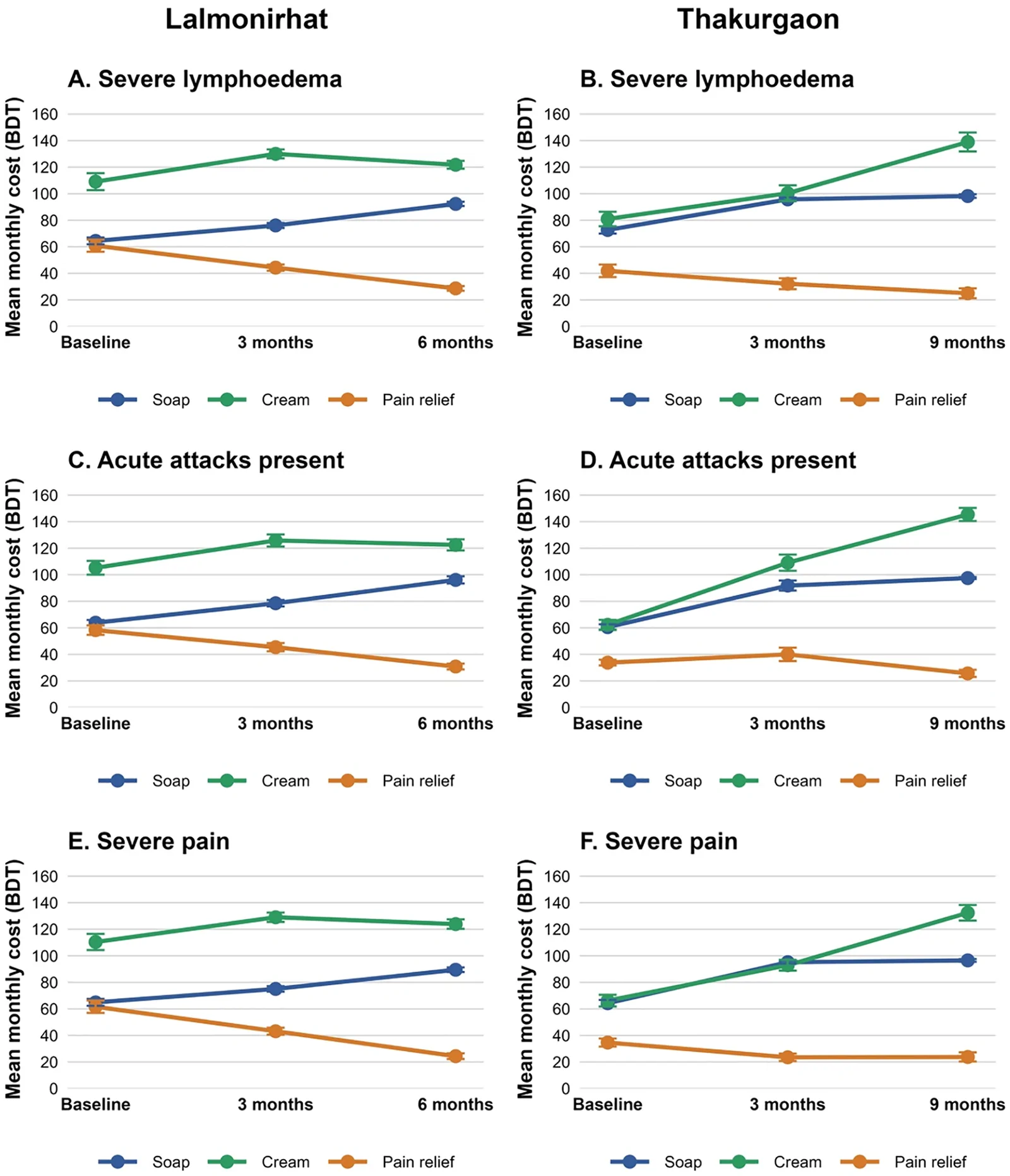
Mean monthly expenditure on self-care items among participants with severe lymphoedema, acute attacks and severe pain at baseline, 3 months and final follow-up, by district. Note: Values represent mean monthly expenditure in Bangladeshi Taka (BDT) on soap, cream and pain relief. Panels A and B show participants with severe lymphoedema; panels C and D show participants reporting acute attacks; and panels E and F show participants reporting severe pain. Final follow-up occurred at 6 months in Lalmonirhat and 9 months in Thakurgaon. Error bars represent standard errors. Baseline statistical comparisons by clinical category are presented in S1 and S2 Tables.

#### Lymphoedema stage.

In Lalmonirhat, mean total monthly self-care expenditure increased across baseline lymphoedema stages, from 159.2 BDT among participants with mild lymphoedema to 197.1 BDT among those with moderate lymphoedema and 234.3 BDT among those with severe lymphoedema (p = 0.003). Expenditure on cream also increased with lymphoedema severity, from 62.5 BDT in the mild group to 94.2 BDT in the moderate group and 109.1 BDT in the severe group (p = 0.009). Corresponding mean pain-relief expenditure was 37.5, 44.5 and 60.8 BDT, respectively (p = 0.014). Soap expenditure did not differ significantly by lymphoedema stage (p = 0.266) (S1 Table).

In Thakurgaon, mean total monthly self-care expenditure was 172.0 BDT among participants with mild lymphoedema, 212.7 BDT among those with moderate lymphoedema and 234.2 BDT among those with severe lymphoedema (p < 0.001). Soap, cream and pain-relief expenditure also differed significantly across lymphoedema-stage categories (all p < 0.001), whereas miscellaneous expenditure did not differ significantly by stage (p = 0.321) (S2 Table).

#### Acute attacks.

In Lalmonirhat, participants reporting acute attacks had a higher mean total monthly self-care expenditure than those not reporting attacks: 227.4 BDT compared with 170.0 BDT (p < 0.001). Participants reporting attacks also had higher mean expenditure on soap (63.9 versus 54.2 BDT; p = 0.015), cream (105.2 versus 80.0 BDT; p < 0.001) and pain relief (58.3 versus 33.2 BDT; p < 0.001) (S1 Table).

In Thakurgaon, mean total monthly expenditure was 174.3 BDT among participants reporting acute attacks and 231.0 BDT among those not reporting attacks (p < 0.001). Participants reporting attacks had higher pain-relief expenditure than those without attacks (33.8 versus 18.6 BDT; p < 0.001), but lower soap expenditure (60.5 versus 74.5 BDT; p = 0.003) and lower miscellaneous expenditure (21.7 versus 80.2 BDT; p < 0.001). Cream expenditure did not differ significantly according to acute-attack status (p = 0.429) (S2 Table).

#### Pain level.

In Lalmonirhat, mean total monthly expenditure was 189.1 BDT among participants reporting mild pain, 189.0 BDT among those reporting moderate pain and 237.0 BDT among those reporting severe pain (p = 0.003). The mild-pain category included only three participants and should therefore be interpreted cautiously. Cream expenditure increased from 66.7 BDT in the mild-pain group to 88.3 BDT in the moderate-pain group and 110.4 BDT in the severe-pain group (p = 0.020). Pain-relief expenditure was 40.0, 42.8 and 61.6 BDT, respectively (p = 0.008). Soap expenditure did not differ significantly by pain level (p = 0.152) (S1 Table).

In Thakurgaon, mean total monthly expenditure was 182.0 BDT among participants reporting mild pain, 200.8 BDT among those reporting moderate pain and 192.1 BDT among those reporting severe pain. Total expenditure did not differ significantly across pain categories (p = 0.565). Pain-relief expenditure increased across the three categories from 18.7 to 24.9 and 34.6 BDT (p = 0.004), while miscellaneous expenditure was 61.4, 55.3 and 30.8 BDT, respectively (p = 0.008). Differences in soap and cream expenditure were not statistically significant (p = 0.428 and p = 0.071, respectively) (S2 Table).

#### Multivariable mixed-effects analysis.

In the exploratory linear mixed-effects analysis, monthly self-care expenditure was higher at 3 months and final follow-up than at baseline in Lalmonirhat (adjusted cost ratio [aCR] 1.13 at both time points; both p < 0.001). At baseline, expenditure was lower in Thakurgaon (aCR 0.82, 95% CI 0.76–0.88; p < 0.001). Significant district-by-time interactions indicated that expenditure increased more strongly over follow-up in Thakurgaon than in Lalmonirhat.

Greater baseline lymphoedema severity was also associated with higher adjusted expenditure (moderate vs mild: aCR 1.13, 95% CI 1.06–1.20; severe vs mild: aCR 1.29, 95% CI 1.21–1.37; both p < 0.001). Age, sex and previous exposure to self-care training were not independently associated with expenditure in the primary model. Full model estimates and model-comparison statistics are presented in S3 Table.

### Objective 4. Relationship between adherence to the enhanced self-care protocol and self-care expenditure

Overall, there was no observed difference by sex, age, or marital status between those who fully adhered and those who partially adhered to the protocol. In Lalmonirhat, 80.5% (120/149) and 61.5% (88/143) of participants reported that they had fully adhered to the enhanced self-care protocol at 3 months and 6 months after the training. In Thakurgaon, 71.4% (95/133) and 60% (75/125) participants reported that they had fully adhered to the enhanced self-care protocol at 3 months and 9 months after the training. Data for each district are represented graphically in S1 Fig to visually highlight the differences.

#### Overall costs.

Mean monthly total self-care expenditure did not differ significantly between fully and partially adherent participants in either district. In Thakurgaon, miscellaneous expenditure was lower among fully adherent participants at both follow-up assessments (Table 4).

**Table 4:** Mean monthly self-care expenditure (BDT) by adherence status, district and follow-up time points.

Item	Stat.	Lalmonirhat District				Thakurgaon District			
		3 months		6 months		3 months		9 months	
Fully Adherent	Partially-adherent	Fully Adherent	Partially-adherent	Fully Adherent	Partially-adherent	Fully Adherent	Partially-adherent
**Soap**	mean	72.3	69.3	89.1	90.5	94.8	95.4	95.6	95.6
	sd	16.5	17.0	13.5	11.3	8.5	6.5	6.2	9.1
**Cream**	mean	119.2	128.2	111.9	115.8	92.1	91.6	123.7	127.2
	sd	32.9	28.2	27.2	25.3	27.8	29.1	38.8	34.1
**Pain relief**	mean	40.0	38.6	24.5	26.4	22.1	25.7	20.7	15.6
	sd	20.6	18.8	15.4	15.1	17.1	16.9	16.7	15.0
**Miscellaneous**	mean	–	–	–	–	27.5	38.9	0.0	3.0
	sd	–	–	–	–	41.7	42.7	0.0	21.2
**Total**	**mean**	**231.5**	**236.1**	**225.6**	**232.7**	**236.5**	**251.6**	**240.0**	**241.4**
	**sd**	**45.8**	**40.9**	**40.1**	**31.9**	**54.3**	**56.6**	**50.0**	**46.9**

BDT = Bangladeshi Taka. Note: Values are mean monthly expenditure and standard deviation (SD). Fully adherent participants performed all five protocol activities daily: cleaning, applying cream, raising or elevating the limb, exercise, and massage. Participants who did not perform all five activities daily were classified as partially adherent. Miscellaneous expenditure was not collected in Lalmonirhat. Total expenditure excludes clinic-visiting costs.

Table 4 shows the average self-care spending (BDT) for participants who fully adhered to the self-care protocol and those who were partially adherent at the 3-month and 6- to 9-month follow-ups.

#### Exploratory mixed-effects logistic regression analysis of adherence.

In the exploratory mixed-effects logistic regression analysis, 506 follow-up observations from 267 participants were included. Comparison of models with and without a district-by-time interaction showed no improvement in fit after inclusion of the interaction (AIC 628.1 vs 627.0; likelihood-ratio test χ^2^ = 0.99, df = 1, p = 0.319). The more parsimonious model without the interaction was therefore retained as the preferred model. In this model, the odds of full adherence were lower at final follow-up than at 3 months (aOR 0.45, 95% CI 0.30–0.67; p < 0.001). District, baseline lymphoedema stage, sex and previous exposure to self-care training were not independently associated with full adherence. Increasing age showed borderline evidence of lower odds of full adherence (aOR 0.80 per 10-year increase, 95% CI 0.63–1.01; p = 0.055). Full model estimates and model-comparison statistics are presented in S4 Table.

## Discussion

This study highlights that even in a LF post-elimination setting, routine self-care for lymphoedema continues to impose measurable out-of-pocket costs on affected households, particularly among those with severe disease. Both Lalmonirhat and Thakurgaon participants exhibited broadly similar trends in the types of items purchased and in how their spending patterns evolved over time. While enhanced self-care was associated with improved clinical outcomes and a shift toward spending on preventative measures (e.g., soap and cream), the total monthly costs are likely to represent a potential financial burden, particularly given that Lalmonirhat and Thakurgaon are among the most socioeconomically disadvantaged districts in Bangladesh [16,17]. Since income data were not collected, these results should be interpreted with caution. The adjusted analyses were consistent with the descriptive findings and indicated that higher expenditure was associated with greater baseline lymphoedema severity and varied over time and between districts. These findings reinforce the disproportionate financial burden experienced by individuals with more advanced disease.

The sensitivity analysis produced similar estimates for the principal associations, although a small positive association with previous self-care training emerged after exclusion of one influential observation. Overall, disease severity and contextual factors appeared to be more important determinants of self-care costs than the demographic characteristics measured in this study. These findings underscore the importance of integrating affordability considerations into MMDP planning, including ensuring reliable access to essential self-care items for individuals with advanced disease [23]. Community-based self-care programmes may further improve programme cost-effectiveness [24]. However, additional assessments of the costs, effectiveness, and wider economic implications of MMDP strategies are needed to more fully evaluate the benefits of the Global Programme to Eliminate Lymphatic Filariasis [25,26].

### Impact of enhanced self‑care protocols

Implementation of the enhanced self‑care protocol was associated with reductions in acute attacks and pain levels, indicating positive clinical and functional outcomes. These findings are consistent with recent enhanced-care studies in Bangladesh and Ethiopia [3,4], and a wide range of studies on lymphoedema and basic hygiene‑based self‑care [27–30]. Embedding such interventions within national programmes aligns with the WHO MMDP guidelines [1] and supports the WHO NTD Roadmap 2021–2030 objectives [19].

Changes in self-care spending following the enhanced self-care protocol suggest a shift towards greater investment in recommended self-care practices. Participants reported higher spending on soap and cream, which may reflect increased engagement with behaviours that improve skin integrity and physical comfort [29]. Simultaneously, reduced spending on pain relief corresponded with lower reported pain and fewer acute attacks, further supporting the potential benefits of the enhanced self-care protocol [3,4]. Together, these findings suggest that participants increasingly prioritised essential self-care items as their symptoms improved. They also reinforce evidence that low-cost, behaviourally supported interventions can improve physical function, productivity and psychosocial wellbeing [27–30]. However, further research evaluating the feasibility and acceptability of implementing the additional components of the enhanced self-care protocol (e.g., self-deep breathing, lymphatic massage, hydration and nutrition) would help determine whether further benefits can be achieved, particularly for older individuals and those with severe lymphoedema [31]. Future studies using a controlled design to compare standard WHO-recommended self-care with the enhanced protocol would help determine the additional economic benefits attributable to the enhanced components.

### Economic burden and spending behaviour

Even at modest levels, self-care costs represent a potential and ongoing financial pressure for affected households. People living with lymphoedema consistently reported paying out‑of‑pocket for soap and antibacterial and/or antifungal creams, the same categories identified as key expenditures in other endemic countries [6,11–13]. In low‑income rural settings like Lalmonirhat and Thakurgaon districts, where healthcare spending is largely financed by households, these costs may become difficult to sustain over time and can negatively impact community-level economic participation and productivity [30,32,33]. Although household-level income data were not collected in this study, the findings should be interpreted alongside national income estimates, which indicate that recurring costs can represent a burden for rural households in Bangladesh [34,35]. Although overall monthly spending did not change significantly, the composition of expenses did. Increased investment in soap and cream after the intervention suggests improved prioritisation of preventive items, while decreased spending on pain relief reflects improved clinical management. These patterns indicate that when participants are equipped with knowledge, and self-care skills they can reallocate limited resources toward more preventive practice. Although access to affordable self-care supplies remains important, health seeking behaviour and level of adherence are likely to be influenced by multiple interacting factors [31,36–39]. The exploratory mixed-effects analysis supported the descriptive findings. After accounting for repeated observations within participants, expenditure was associated with follow-up time, district and greater baseline lymphoedema severity.

The substantially improved fit of the model including the district-by-time interaction supported differences in expenditure trajectories between the two districts. Individuals with more severe lymphoedema consistently had higher adjusted expenditure. In the primary interaction model, age, sex and previous exposure to self-care training were not independently associated with expenditure. This suggests that sustained adherence may be influenced by factors not captured in this dataset, such as household circumstances, access to supplies, physical capacity, social support or competing demands. The borderline association between increasing age and lower odds of full adherence should be interpreted cautiously and requires confirmation in larger studies.

While acute attacks decreased substantially during follow-up, overall monthly expenditure remained broadly stable. This appears to reflect a shift in spending patterns rather than an overall reduction in financial burden, with participants spending less on pain-relief medications and more on preventative self-care items such as soap and creams. Consequently, although improved management may have reduced symptom-related expenditures, the economic benefit of fewer acute attacks could not be quantified directly within this study. Future research should evaluate whether reductions in acute attacks translate into broader economic benefits, including reduced healthcare expenditures, improved productivity and decreased household economic burden. The study measured only expenditure on self-care items and did not collect information on healthcare utilisation, transport costs, work days lost, caregiver burden, or productivity losses. Therefore, the wider economic consequences of reduced acute attacks could not be assessed.

The lack of a cost difference between fully and partially adherent participants suggests that factors beyond expenditure may contribute to adherence behaviours. It may also reflect the fact that even those with lower adherence still need to purchase essential items. Expenditure patterns may be influenced by the frequency and amount of use; for example, a larger area of an affected leg may require more cream, or an individual with more acute attacks may require more pain relief. These clinical factors need further consideration. In addition, future studies should include more specific information on footwear-related expenditures, as they are part of the MMDP strategies and were not collected separately. It would also be beneficial to gather information on whether the affected individuals require modified footwear, and the associated costs. An important finding from the adherence analysis was the decline in full adherence between the 3-month and final follow-up assessments. Although uptake of the enhanced self-care protocol was high shortly after training, maintaining daily self-care behaviours over longer periods appeared more challenging. This finding has important programmatic implications and suggests that initial training alone may not be sufficient to sustain long-term behavioural change. Refresher training, periodic follow-up visits, peer-support mechanisms and community-based reinforcement strategies may help maintain adherence and preserve the clinical improvements associated with self-care practices. Future studies should investigate the most effective approaches for supporting long-term adherence within national MMDP programmes.

### Inequities in disease burden and support

Costs increased with disease severity, particularly among participants experiencing acute attacks or persistent pain, highlighting the disproportionate burden faced by those with advanced disease. Similar patterns have been reported elsewhere, where greater acute attacks and chronic disease associated with higher out‑of‑pocket expenditure, reduced productivity, and reliance on family support [7,11–13]. Such economic vulnerability and strain can exacerbate poverty cycles for households and communities, especially where chronic illness limits income‑earning capacity for the person affected and their caregiver [14,15,32,33].

The observed reduction in acute attacks among participants using the enhanced self-care protocol may also have contributed to improved wellbeing and daily functioning by reducing pain, physical discomfort, and disruption to daily living activities [30,40–42]; however, quality of life was not directly measured in this study. There is a need to better understand how MMDP affects physical, psychological, and social aspects of life and whether the economic pressures associated with ongoing self-care negatively impact wellbeing, even when clinical symptoms improve. Strengthening social support systems and integrating financial or material assistance, for example, subsidised soap or cream, could improve adherence and reduce the cumulative stress of long‑term disease management [28–31]. Expanding and systematically monitoring these measures across all areas with a history of LF morbidity [6,43] is essential to ensure equitable access and maximise public health benefit. This will also help to meet key WHO NTD road map goals and ensure no one is left behind [19].

### Limitations and strengths

This study has several limitations. It relied on self-reported adherence data, which may be subject to recall and social desirability bias [44,45] and may have led to overestimation of adherence.

In addition, the binary classification of adherence may have obscured important variation in the frequency of individual self-care activities. Future studies should examine activity-specific adherence patterns and barriers to sustained implementation of individual self-care behaviours. The study sample was drawn from two high‑endemic, low‑income districts in northern Bangladesh, which may limit generalisability as the out-of-pocket expenses may not fully capture variation in access or affordability across other endemic areas. The absence of a concurrent control group limits the ability to infer causality. Although improvements in clinical outcomes and expenditure patterns were observed following enhanced self-care training, these changes may also have been influenced by seasonal variation, regression to the mean, district-level contextual factors, or other unmeasured influences. Previous cluster randomized controlled trials comparing enhanced self-care and standard self-care, showed improvements in lymphoedema severity, mid-calf tissue compressibility and acute attacks [3,4]. The final follow-up period was limited to 6 and 9 months, which may not fully capture long‑term adherence or economic adaptation; future studies should consider a longer follow-up period of 1–2 years or annual assessments. The adherence model was exploratory and relied on self-reported frequency of self-care activities. The classification also required complete responses for all five activities. The study did not collect household income or other potentially important socioeconomic and behavioural determinants, including access to water and self-care supplies, caregiving or household support. Consequently, the economic burden on affected individuals and families could not be fully assessed, and residual confounding in the exploratory multivariable analyses remains possible. The mixed-effects analyses were not prespecified and should therefore be interpreted as hypothesis-generating rather than confirmatory. Self-reported costs may also be affected by recall error and should be interpreted as approximate measures of routine self-care expenditure. Further, detailed information on self-care practices prior to training was not collected. As a result, behavioural changes following the intervention could not be quantified directly and pre-existing self-care behaviours may have influenced observed outcomes. However, recent LF MMDP training activities in the study areas had been limited and were therefore unlikely to have substantially influenced the results. Nevertheless, this limitation should be considered when interpreting the findings. Finally, seasonal variation and contextual factors during the 2020–2021 study period, including the COVID-19 pandemic, may also have influenced health-seeking behaviour and household expenditure patterns.

Nonetheless, the study offers one of the few longitudinal analyses of patient‑level data combining clinical, behavioural, and economic dimensions of lymphoedema self‑care. The inclusion of exploratory mixed-effects analyses strengthened the interpretation of the longitudinal findings by accounting for repeated measurements within participants. It provides a valuable real‑world understanding of how people living with LF manage chronic illness amid financial outlays. Further, it provides a basis for future studies on the economic burden to patients, which are currently lacking. Given the recurring costs associated with recommended self-care practices, national programmes may wish to consider mechanisms to improve affordable or subsidised access to essential self-care supplies, particularly soap and creams for those living with advanced lymphoedema.

Attention to the range of limitations outlined above would be highly beneficial.

## Conclusion

This study highlights the economic and behavioural realities of lymphoedema self-care in resource-limited settings and provides evidence that out-of-pocket spending remains necessary even where LF has been eliminated as a public health problem. Both the descriptive analyses and the exploratory mixed-effects models consistently demonstrated that self-care expenditure was greater among individuals with more severe lymphoedema and varied over time and between districts. Although enhanced self-care was associated with improved clinical outcomes and changes in spending towards preventative self-care practices, ongoing out-of-pocket costs remain a potential barrier to sustained self-care, particularly for those with advanced disease. For Bangladesh’s MMDP programme, these findings highlight the importance of considering affordability and equitable access to essential self-care supplies, particularly for individuals with severe lymphoedema. Future controlled studies are needed to confirm these associations and further evaluate the long-term effectiveness and economic impact of enhanced self-care approaches. In addition, strategies such as refresher training and community-based support should be explored to maintain long-term adherence to self-care practices.

These findings support continued investment in WHO-aligned morbidity management programmes while highlighting the importance of addressing the long-term affordability of self-care as part of universal health coverage and the Sustainable Development Goal 3 agenda.

## Supporting information

S1 Text Standard and enhanced self-care interventions and lymphoedema staging classifications. (DOCX)

S1 Fig Percentage of participants classified as fully or partially adherent at follow-up. (TIFF)

S2 Fig Mean monthly self-care expenditure by item category and assessment time point. (TIFF)

S1 Table Baseline self-care expenditure by clinical characteristics in Lalmonirhat. (DOCX)

S2 Table Baseline self-care expenditure by clinical characteristics in Thakurgaon. (DOCX)

S3 Table Exploratory multivariable linear mixed-effects analysis of monthly self-care expenditure. (DOCX)

S4 Table Exploratory mixed-effects logistic regression analysis of adherence. (DOCX)

S1 Checklist STROBE Checklist. (DOC)
