# Supplementary material for: Evaluating the cost of self-care: Impact of enhanced morbidity management protocol on people living with lymphatic filariasis in Bangladesh
Source: PLoS Negl Trop Dis. 2026 Oct 21;20(10):e0014812. doi: 10.1371/journal.pntd.0014812 (PMC13743021; doi:10.1371/journal.pntd.0014812)
Supplement: Supplementary file 1 [file pntd.0014812.s001.docx]

**S1 Text. Summary of the standard hygiene-based care and enhanced self-care protocol additions and summary of WHO and Dreyer lymphoedema stages**.

Standard hygiene-based care:

- Limb hygiene: washing and drying affected limbs with soap and clean towels twice daily.
- Moisturising: applying antibacterial and/or antifungal cream to reduce skin cracking.
- Limb elevation: elevating legs to reduce swelling during rest.
- Daily limb exercises: to improve lymphatic drainage and range of motion
- Protective footwear: to prevent injuries and infections.
- Prompt management of acute attacks: using prescribed medications, warm compresses and a massage.
- Provision of wash kits (soap, cloths, antibacterial and/or antifungal creams) via community clinics.

Enhanced self-care additions:

- Deep-breathing exercises: to stimulate lymph flow and promote drainage.
- Self-lymphatic drainage massage: gentle massage moving fluid proximally.
- Hydration: drinking clean water regularly.
- Nutrition: consuming fresh fruits and vegetables to support tissue health.

Summary of WHO and Dreyer lymphoedema stages

- Mild (Dreyer Stage 1-2):
  - Reversible swelling (often subsides overnight and with elevation)
  - Skin is soft, with no or minimal skin changes
- Moderate (Dreyer stage 3-4):
  - Persistent swelling harder to reduce
  - Skin thickening begins, often with early signs of fibrosis or skin folds
- Severe (Dreyer Stage 5-7):
  - Extensive and irreversible swelling
  - Skin changes such as nodules, deep folds, papillomatosis.
  - Often associated with secondary infections and reduced mobility.
