# Supplementary material for: Evaluating the cost of self-care: Impact of enhanced morbidity management protocol on people living with lymphatic filariasis in Bangladesh
Source: PLoS Negl Trop Dis. 2026 Oct 21;20(10):e0014812. doi: 10.1371/journal.pntd.0014812 (PMC13743021; doi:10.1371/journal.pntd.0014812)
Supplement: Supplementary file 3 [file pntd.0014812.s005.docx]

**Table S2. Baseline monthly self-care expenditure by clinical characteristics in Thakurgaon district.**

| **Characteristic** | **Category** | **n (%)** | **Soap Mean (SD)** | **p-value** | **Cream Mean (SD)** | **p-value** | **Pain relief Mean (SD)** | **p-value** | **Misc. Mean (SD)** | **p-value** | **Total Mean (SD)** | **p-value** |
| --- | --- | --- | --- | --- | --- | --- | --- | --- | --- | --- | --- | --- |
| **Lymphoedema stage** | Mild | 76 (55.1) | 60.5 (19.0) | <0.001 | 50.3 (31.9) | <0.001 | 23.3 (14.5) | <0.001 | 43.6 (48.1) | 0.321 | 172.0 (63.1) | <0.001 |
|  | Moderate | 30 (21.7) | 71.3 (15.9) |  | 65.0 (31.3) |  | 26.0 (18.3) |  | 58.1 (43.3) |  | 212.7 (61.1) |  |
|  | Severe | 32 (23.2) | 72.7 (15.6) |  | 80.9 (30.7) |  | 41.9 (26.6) |  | 41.3 (42.6) |  | 234.2 (61.6) |  |
| **Acute attacks** | Yes | 87 (63.0) | 60.5 (19.5) | 0.003 | 62.3 (34.5) | 0.429 | 33.8 (19.8) | <0.001 | 21.7 (36.6) | <0.001 | 174.3 (66.8) | <0.001 |
|  | No | 51 (37.0) | 74.5 (12.4) |  | 57.6 (32.5) |  | 18.6 (17.0) |  | 80.2 (34.4) |  | 231.0 (52.4) |  |
| **Pain level** | Mild | 15 (10.9) | 62.0 (15.7) | 0.428 | 44.0 (22.3) | 0.071 | 18.7 (15.1) | 0.004 | 61.4 (44.0) | 0.008 | 182.0 (51.7) | 0.565 |
|  | Moderate | 67 (48.6) | 67.7 (19.0) |  | 59.6 (35.7) |  | 24.9 (17.1) |  | 55.3 (45.8) |  | 200.8 (68.1) |  |
|  | Severe | 56 (40.6) | 64.3 (18.5) |  | 66.2 (32.8) |  | 34.6 (22.8) |  | 30.8 (42.9) |  | 192.1 (70.7) |  |

Note: Values are mean (standard deviation) monthly expenditure in Bangladeshi Taka (BDT). Percentages are based on participants with valid data for each clinical characteristic. p-values are shown once for each overall comparison. One-way analysis of variance was used for lymphoedema stage and pain level; Welch's independent-samples t-test was used for acute attacks. Total expenditure comprised soap, cream and pain-relief expenditure in Lalmonirhat, with miscellaneous expenditure additionally included in Thakurgaon. Clinic-visiting costs were excluded.
