# Supplementary material for: Evaluating the cost of self-care: Impact of enhanced morbidity management protocol on people living with lymphatic filariasis in Bangladesh
Source: PLoS Negl Trop Dis. 2026 Oct 21;20(10):e0014812. doi: 10.1371/journal.pntd.0014812 (PMC13743021; doi:10.1371/journal.pntd.0014812)
Supplement: Supplementary file 4 [file pntd.0014812.s006.docx]

**Table S3. Exploratory multivariable linear mixed-effects models of factors associated with monthly self-care expenditure**

| **Characteristic** | **Model 1** | | | **Model 2** | | |
| --- | --- | --- | --- | --- | --- | --- |
|  | **aCR** | **95% CI** | **p-value** | **aCR** | **95% CI** | **p-value** |
| ***Assessment time*** | | | | | | |
| 3 months vs baseline | 1.23 | 1.17–1.29 | <0.001 | 1.13 | 1.06–1.21 | <0.001 |
| Final follow-up vs baseline | 1.32 | 1.25–1.39 | <0.001 | 1.13 | 1.05–1.21 | <0.001 |
| ***District*** | | | | | | |
| Thakurgaon vs Lalmonirhat | 0.96 | 0.91–1.01 | 0.142 | 0.82 | 0.76–0.88 | <0.001 |
| ***Baseline lymphoedema severity*** | | | | | | |
| Moderate vs mild | 1.13 | 1.06–1.20 | <0.001 | 1.13 | 1.06–1.20 | <0.001 |
| Severe vs mild | 1.29 | 1.21–1.37 | <0.001 | 1.29 | 1.21–1.37 | <0.001 |
| ***Demographic and training covariates*** | | | | | | |
| Age, per 10-year increase | 1.01 | 0.99–1.03 | 0.462 | 1.01 | 0.99–1.03 | 0.492 |
| Female vs male | 1.03 | 0.98–1.09 | 0.183 | 1.03 | 0.98–1.08 | 0.207 |
| Previous self-care training: yes vs no | 1.03 | 0.98–1.08 | 0.209 | 1.03 | 0.98–1.07 | 0.243 |
| ***District × time interaction*** | | | | | | |
| 3 months × Thakurgaon | — | — | — | 1.19 | 1.08–1.31 | <0.001 |
| Final follow-up × Thakurgaon | — | — | — | 1.40 | 1.27–1.54 | <0.001 |
| **Log-likelihood** | **−182.60** |  |  | **−161.01** |  |  |
| **AIC** | **387.20** |  |  | **348.03** |  |  |
| **BIC** | **438.69** |  |  | **408.88** |  |  |

Note: Likelihood-ratio test comparing Model 1 and Model 2: χ²=43.17, df=2, p<0.001.

Model 2 provided substantially better fit and was retained as the preferred expenditure model. Both models included a participant-level random intercept and were fitted to the same 797 observations. aCR = adjusted cost ratio.

The harmonised expenditure outcome comprised monthly spending on soap, cream and pain relief in both districts and was modelled as log(cost + 1). Lalmonirhat, baseline, mild lymphoedema, male sex and no previous self-care training were the reference categories. In Model 2, the time coefficients represent change over time in Lalmonirhat, the district coefficient represents the Thakurgaon–Lalmonirhat difference at baseline, and the interaction terms represent the additional relative change over time in Thakurgaon.
