# Supplementary material for: Evaluating the cost of self-care: Impact of enhanced morbidity management protocol on people living with lymphatic filariasis in Bangladesh
Source: PLoS Negl Trop Dis. 2026 Oct 21;20(10):e0014812. doi: 10.1371/journal.pntd.0014812 (PMC13743021; doi:10.1371/journal.pntd.0014812)
Supplement: Supplementary file 5 [file pntd.0014812.s007.docx]

**Table S4. Exploratory multivariable mixed-effects logistic regression models of factors associated with full adherence**

| **Characteristic** | **Model 1** | | | **Model 2** | | |
| --- | --- | --- | --- | --- | --- | --- |
|  | **aOR** | **95% CI** | **p-value** | **aOR** | **95% CI** | **p-value** |
| ***Follow-up time*** | | | | | | |
| Final follow-up vs 3 months | 0.45 | 0.30–0.67 | <0.001 | 0.36 | 0.20–0.65 | <0.001 |
| ***District*** | | | | | | |
| Thakurgaon vs Lalmonirhat | 0.72 | 0.43–1.19 | 0.195 | 0.57 | 0.29–1.12 | 0.104 |
| ***Baseline lymphoedema severity*** | | | | | | |
| Moderate vs mild | 1.29 | 0.71–2.36 | 0.403 | 1.29 | 0.71–2.36 | 0.402 |
| Severe vs mild | 0.95 | 0.52–1.73 | 0.874 | 0.95 | 0.52–1.73 | 0.870 |
| ***Demographic and training covariates*** | | | | | | |
| Age, per 10-year increase | 0.80 | 0.63–1.01 | 0.055 | 0.80 | 0.63–1.01 | 0.057 |
| Female vs male | 0.84 | 0.51–1.36 | 0.472 | 0.84 | 0.51–1.36 | 0.474 |
| Previous self-care training: yes vs no | 0.89 | 0.57–1.38 | 0.597 | 0.89 | 0.57–1.38 | 0.596 |
| ***District × time interaction*** | | | | | | |
| Final follow-up × Thakurgaon | — | — | — | 1.51 | 0.67–3.37 | 0.319 |
| **Log-likelihood** | **−304.52** |  |  | **−304.03** |  |  |
| **AIC** | **627.05** |  |  | **628.05** |  |  |
| **BIC** | **665.09** |  |  | **670.32** |  |  |

Note: Likelihood-ratio test comparing Model 1 and Model 2: χ²=0.99, df=1, p=0.319.

Model 1 included main effects only; Model 2 additionally included a district x time interaction.

Model 1 provided the more parsimonious fit and was retained as the preferred adherence model. Both models included a participant-level random intercept and were fitted to the same 506 observations. aOR = adjusted odds ratio.

Full adherence was defined as reported daily performance of all five recommended self-care activities: cleaning, applying cream, raising the affected limb, exercise and massage. The analysis included the 3-month and final follow-up assessments (6 months in Lalmonirhat and 9 months in Thakurgaon). Lalmonirhat, the 3-month assessment, mild lymphoedema, male sex and no previous self-care training were the reference categories.
