# Supplementary material for: Evaluating the cost of self-care: Impact of enhanced morbidity management protocol on people living with lymphatic filariasis in Bangladesh
Source: PLoS Negl Trop Dis. 2026 Oct 21;20(10):e0014812. doi: 10.1371/journal.pntd.0014812 (PMC13743021; doi:10.1371/journal.pntd.0014812)
Supplement: Supplementary file 6 [file pntd.0014812.s008.doc]

STROBE Statement—Checklist of items that should be included in reports of ***cohort studies***

|  | Item No | Recommendation |
| --- | --- | --- |
| **Title and abstract** | 1 | (*a*) A longitudinal cohort study cohort was conducted… |
|  |  | (*b*) Abstract – Background, methodology, Results, Conclusion |
| Introduction | | |
| Background/rationale | 2 | Introduction paragraphs 1-5 |
| Objectives | 3 | End of Introduction – Objectives section |
| Methods | | |
| Study design | 4 | Methods – Study Design |
| Setting | 5 | Methods – Setting section |
| Participants | 6 | (*a*) Methods – Population and Sample Size; Data Collection and Variables |
|  |  | (*b*) Methods – Study design; Results – Summary of study period |
| Variables | 7 | Methods – Data Collection and Variables |
| Data sources/ measurement | 8* | Methods – Data Collection and Variables |
| Bias | 9 | Discussion - Limitations |
| Study size | 10 | Methods – Population and Sample Size |
| Quantitative variables | 11 | Methods – Data Analysis |
| Statistical methods | 12 | (*a*) Statistical methods – Data Analysis, including Exploratory multivariable analyses |
|  |  | (*b*) Subgroup interactions |
|  |  | (*c*) Methods – Data Analysis |
|  |  | (*d*) Results – Summary of study period and participant characteristics |
|  |  | (*e*) Methods – Data Analysis |
| Results | | |
| Participants | 13* | (a) Results – Summary of study period, Tables 2 and 3 |
|  |  | (b) Results – Summary paragraph |
|  |  | (c) Participants flow described in Results text and Tables 2 and 3 |
| Descriptive data | 14* | (a) Results Table 1 |
|  |  | (b) Methods – Data Analysis and Results tables. |
|  |  | (c) Summarise follow-up time (eg, average and total amount) |
| Outcome data | 15* | Results – Tables 2-4 |
| Main results | 16 | (*a*) Results – Objectives 2 and 3 sections |
|  |  | (*b*) Methods – Lymphoedema Stage definitions |
|  |  | (*c*) n/a |
| Other analyses | 17 | Results – Objective 4; Exploratory multivariable analyses (Supplementary Tables S3 and S4) |
| Discussion | | |
| Key results | 18 | Discussion – first paragraph |
| Limitations | 19 | Discussion – Limitations and Strengths section |
| Interpretation | 20 | Discussion section |
| Generalisability | 21 | Discussion – Limitations section |
| Other information | | |
| Funding | 22 | n/a |

*Give information separately for exposed and unexposed groups.

**Note:** An Explanation and Elaboration article discusses each checklist item and gives methodological background and published examples of transparent reporting. The STROBE checklist is best used in conjunction with this article (freely available on the Web sites of PLoS Medicine at http://www.plosmedicine.org/, Annals of Internal Medicine at http://www.annals.org/, and Epidemiology at http://www.epidem.com/). Information on the STROBE Initiative is available at http://www.strobe-statement.org.
